# Early Insights from Statistical and Mathematical Modeling of Key Epidemiologic Parameters of COVID-19

**DOI:** 10.3201/eid2611.201074

**Published:** 2020-11

**Authors:** Matthew Biggerstaff, Benjamin J. Cowling, Zulma M. Cucunubá, Linh Dinh, Neil M. Ferguson, Huizhi Gao, Verity Hill, Natsuko Imai, Michael A. Johansson, Sarah Kada, Oliver Morgan, Ana Pastore y Piontti, Jonathan A. Polonsky, Pragati Venkata Prasad, Talia M. Quandelacy, Andrew Rambaut, Jordan W. Tappero, Katelijn A. Vandemaele, Alessandro Vespignani, K. Lane Warmbrod, Jessica Y. Wong, for the WHO COVID-19 Modelling Parameters Group

**Affiliations:** Centers for Disease Control and Prevention, Atlanta, Georgia, USA (M. Biggerstaff, M.A. Johansson, S. Kada, P.V. Prasad, T.M. Quandelacy);; University of Hong Kong, Hong Kong, China (B.J. Cowling, H. Gao, J.Y. Wong);; Imperial College London, London, UK (Z.M. Cucunubá, N.M. Ferguson, N. Imai);; World Health Organization, Geneva, Switzerland (L. Dinh, O. Morgan, J.A. Polonsky, J.W. Tappero, K.A. Vandemaele, K.L. Warmbrod);; University of Edinburgh, Edinburgh, Scotland, UK (V. Hill, A. Rambaut);; Northeastern University, Boston, Massachusetts, USA (A. Pastore y Piontti, A. Vespignani);; ISI Foundation, Turin, Italy (A. Vespignani)

**Keywords:** COVID-19, epidemiological parameters, mathematical modeling, World Health Organization, coronavirus, viruses, 2019 novel coronavirus disease, SARS-CoV-2, severe acute respiratory syndrome coronavirus 2, zoonoses

## Abstract

We report key epidemiologic parameter estimates for coronavirus disease identified in peer-reviewed publications, preprint articles, and online reports. Range estimates for incubation period were 1.8–6.9 days, serial interval 4.0–7.5 days, and doubling time 2.3–7.4 days. The effective reproductive number varied widely, with reductions attributable to interventions. Case burden and infection fatality ratios increased with patient age. Implementation of combined interventions could reduce cases and delay epidemic peak up to 1 month. These parameters for transmission, disease severity, and intervention effectiveness are critical for guiding policy decisions. Estimates will likely change as new information becomes available.

On December 31, 2019, authorities in China notified the World Health Organization (WHO) of a pneumonia cluster of unknown etiology in Wuhan (*1*); a novel coronavirus was subsequently isolated. As of March 7, 2020, coronavirus disease (COVID-19) and its causative agent, severe acute respiratory syndrome coronavirus 2 (SARS-CoV-2), had resulted in 101,927 cases and 3,486 deaths in 94 countries spanning 6 continents (*2*). The spectrum of illness ranged from asymptomatic infection to mild disease (e.g., fever, dry cough, and myalgias), pneumonia, and death. Roughly 20% of cases require hospitalization for shortness of breath; death is associated with increasing age and underlying conditions (e.g., hypertension, cardiovascular disease, and diabetes) (*3*).

We review major parameters of COVID-19 transmission dynamics from statistical and mathematical modeling studies using epidemiologic data reported in the first 60 days of the epidemic. We estimate the key components that contribute to future modeling on the effects of nonpharmaceutical interventions (NPIs) and to inform critical resource allocation decisions (*4*). Data estimates were current as of March 6, 2020, a few days before WHO characterized COVID-19 as a pandemic on March 11, 2020 (WHO Director General remarks, https://www.youtube.com/watch?v=sbT6AANFOm4), and were subject to change as more information becomes available.

## Methods and Results

We reviewed the literature on key epidemiologic parameters (Table 1) relating to the COVID-19 epidemic. This report is not a formal systematic review because the epidemic is rapidly unfolding and useful data sources exist that have not yet been peer reviewed. We searched the peer-reviewed and gray literature, including preprints, research reports, and forum posts. We conducted searches for individual parameters during February 25–March 6, 2020, on PubMed, medRxiv, bioRxiv, arXiv, SSRN, Research Square, Virological, Imperial College COVID reports, and Wellcome Open Research. Search terms centered on the various names of the disease and virus over the course of the epidemic (“nCoV,” “COVID,” “SARS-CoV-2,” “novel coronavirus”) and keywords relating to each of the epidemiologic parameters or characteristics considered (Appendix Table 1). We selected genetic epidemiology estimates, such as evolutionary rate and time from last common ancestor, from Virological (http://virological.org). We included articles in English and Chinese if they used mathematical or statistical methods for adjustment of different biases and if they were either peer reviewed or non–peer reviewed requiring established methods (i.e., clarity about the data used, known statistical methods, and reported uncertainty) (*5*–*8*).

**Table 1:** Key parameters and definitions for modeling of coronavirus disease

Parameter	Definition
Basic reproduction number (R_0_)	Average number of persons infected by a single infected individual in a fully susceptible population
Time-varying or effective reproduction number (R, R_t_, R_E_)	Average number of persons infected by an infected individual in a population in the context of changing transmission patterns, such as those resulting from interventions and acquired immunity
Incubation period	Time between infection and symptom onset
Serial interval	Average time between symptom onset in a primary case and symptom onset in linked secondary cases
Generation interval	Average time between infection of a primary case and infection of linked secondary cases
Doubling time	Average time for the daily case count to double
Infectious period	Period during which an infected host, with or without symptoms, can transmit an infectious agent to susceptible persons, directly or indirectly
Case-fatality ratio	Proportion of cases that result in death (with case defined in numerous ways)
Infection-fatality ratio	Proportion of all infections (confirmed, symptomatic, asymptomatic) that result in death
Mean evolutionary rate	Average rate at which mutations accumulate per base pair in the genome over the course of a year

For each parameter, characteristics such as study population, assumptions, and analytical methods were summarized when patterns were discernible across estimates. Estimates were summarized as ranges to reflect remaining uncertainty. No meta-analyses were performed.

### R_0_ and R

**Figure 1:**
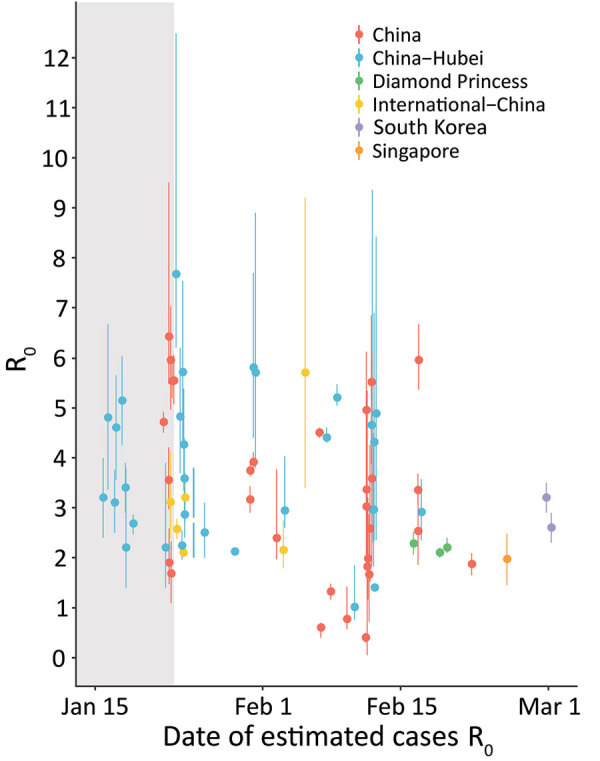
Basic reproduction number (R_0_) estimates for coronavirus disease by date of last reported cases analyzed and location. Points are mean or median estimates and error bars indicate 90% (*12*,*13*,*15*) or 95% bounds (i.e., confidence or credible intervals). International–China estimates are those using international cases or exported cases from China to infer R_0_ in China or Hubei Province. Estimates for China refer to R_0_ estimates at the national or province level, except for those exclusive estimating R_0_ for Hubei (China–Hubei). The gray shaded bar represents the time period before January 23, 2020, the date when broad restrictions were implemented in Hubei Province.

One of the key early indicators of transmissibility of a novel pathogen is R_0_, the basic reproduction number, which represents the average number of persons infected by an incident person in a fully susceptible population. Values for R_0_ that are >1 are considered a critical threshold for epidemic growth. Mean R_0_ estimates for Hubei Province, China, ranged widely, 2.1–5.1 (peer-reviewed) and 2.0–7.7 (M.S. Majumder and K.D. Mandl, unpub. data, https://papers.ssrn.com/sol3/papers.cfm?abstract_id=3524675; T. Liu et al., unpub. data, https://www.biorxiv.org/content/10.1101/2020.01.25.919787v2; K. Mizumoto et al., unpub. data, https://www.medrxiv.org/content/10.1101/2020.02.12.20022434v2.full.pdf; C. Zhou, unpub. data, https://www.medrxiv.org/content/10.1101/2020.02.15.20023440v2.full.pdf; H. Sun et al., unpub. data, https://www.medrxiv.org/content/10.1101/2020.02.17.20024257v1; J. Li et al., unpub. data, https://www.medrxiv.org/content/10.1101/2020.02.18.20024315v1.full.pdf; S. Zhao et al, unpub. data, https://www.medrxiv.org/content/10.1101/2020.02.26.20028449v1.full.pdf; S. Zhao et al., unpub. data, https://doi.org/10.2139/ssrn.3543150; Z. Zhuang et al., unpub. data, https://www.medrxiv.org/content/10.1101/2020.03.02.20030312v1.full.pdf; S. Zhao et al., unpub. data, https://www.medrxiv.org/content/10.1101/2020.02.21.20026559v1), reflecting a variety of assumptions and methods used and data uncertainty (*9*–*17*). A subset of more recent estimates accounted for the broad restrictions implemented on January 23 in Hubei explicitly and were lower than earlier estimates (1.0–2.9) (H. Sun et al., unpub. data, https://www.medrxiv.org/content/10.1101/2020.02.17.20024257v1; L. Xu et al., unpub. data, https://www.medrxiv.org/content/10.1101/2020.02.25.20024398v1; L. Zhang et al., unpub. data, https://www.medrxiv.org/content/10.1101/2020.02.16.20023804v1). Mean R_0_ estimates for provinces outside Hubei or for all of China were similar to those for Hubei before the implementation of travel restrictions (peer-reviewed range 0.4–3.9, preprint range: 0.6–6.4) (*11*,*14*,*18*–*20*; T. Liu et al., unpub. data, https://www.biorxiv.org/content/10.1101/2020.01.25.919787v2; H. Sun et al., unpub. data, https://www.medrxiv.org/content/10.1101/2020.02.17.20024257v1; L. Xu et al., unpub. data, https://www.medrxiv.org/content/10.1101/2020.02.25.20024398v1; L. Tindale et al., unpub. data, https://www.medrxiv.org/content/10.1101/2020.03.03.20029983v1; M. Shen et al., unpub. data, https://www.biorxiv.org/content/10.1101/2020.01.23.916726v1; L. Wang et al., unpub. data, https://www.medrxiv.org/content/10.1101/2020.02.29.20029421v1.full.pdf; Ku et al., unpub. data, http://dx.doi.org/10.2139/ssrn.3543589). R_0_ estimates for China and cases outside China attributed to exportation were generally lower (peer-reviewed range 2.1–3.2; preprint range 2.1– 5.7) (*14*,*15*,*21*; J.M. Read et al., unpub. data, https://www.medrxiv.org/content/10.1101/2020.01.23.20018549v2.full.pdf; C. Zhang and M.Wang, unpub. data, https://www.biorxiv.org/content/10.1101/2020.01.25.919688v3; Q. Zhou et al., unpub. data, https://www.medrxiv.org/content/10.1101/2020.02.06.20020941v1.full.pdf; Volz et al., unpub. data, https://spiral.imperial.ac.uk/bitstream/10044/1/77169/11/2020-02-15-COVID19-Report-5.pdf; Q. Zhao et al., unpub. data, https://www.medrxiv.org/content/10.1101/2020.02.06.20020941v1), as were estimates for the Diamond Princess cruise ship (mean R_0_ »2.2) (*22*; S. Zhao, unpub. data [R_0_ = 2.1], https://doi.org/10.2139/ssrn.3543150; S. Zhao, unpub. data [R_0_ = 2.2], https://www.medrxiv.org/content/10.1101/2020.02.26.20028449v1.full.pdf) and estimates for Singapore and South Korea (range 2.6–3.2; L. Tindale et al., unpub. data, https://www.medrxiv.org/content/10.1101/2020.03.03.20029983v1; Z. Zhuang, unpub. data [R_0_ = 2.6], https://www.medrxiv.org/content/10.1101/2020.03.02.20030312v1.full.pdf) were generally lower. A meta-analysis of 7 early COVID-19 studies that accounted for uncertainty in assumptions estimated an R_0_ of 2.9 (95% CI 2.1–4.5; S.W. Park et al., unpub. data, https://www.medrxiv.org/content/10.1101/2020.01.30.20019877v4) (Figure 1).

High variability in R_0_ estimates can result from a mix of data (e.g., time period of cases analyzed, data available by onset date), methods (e.g., R_0_ as a component of early exponential growth, fitting case data to compartmental models), and assumptions (e.g., serial intervals, case ascertainment). In particular, serial interval estimates directly affect R_0_: shorter serial intervals suggest that fewer transmission events are required for rapid growth. However, most R_0_ estimates reviewed here used serial intervals values between 7.5 (COVID-19) and 8.4 (SARS); these differences likely had limited effects (*7*,*9*).

R_0_ reflects average transmission, not individual-level transmission. Variability (dispersion) among individual-level contacts and transmission potential can lead to many persons infecting no others, whereas some infect many, as previously observed for severe acute respiratory syndrome (SARS) and Middle East respiratory syndrome (MERS) (*23*,*24*). This pattern has also been observed for COVID-19, with estimates of the dispersion parameter <1 (e.g., 0.5 in Singapore [A. Tariq et al. unpub. data, https://www.medrxiv.org/content/10.1101/2020.02.21.20026435v4.full.pdf], 0.54 in China [*14*], 0.58 in Shenzhen [*19*]). These findings imply that a minority of cases may cause the majority of infections; for example, in Shenzhen, 8.9% of cases were found to cause 80% of infections (*19*). Rigorous contact tracing data are needed to improve these estimates and identify opportunities to tailor interventions accordingly (*25*).

Explicit estimates of the time-varying or effective reproducton number, R (often referred to as R_t_ or R_E_), can identify changes in transmission over time as a result of interventions and acquired immunity. Mean estimates of R before January 23 generally fall within the ranges of 2.3–2.6 (peer-reviewed [*26*,*27*]) and 3.9–6.2 (preprints; T. Liu et al., unpub. data, https://www.biorxiv.org/content/10.1101/2020.01.25.919787v2; C. Wang et al., unpub. data, https://www.medrxiv.org/content/10.1101/2020.03.03.20030593v1). Shortly after the travel restrictions, R estimates ranging from 0.4–1.0 (peer-reviewed) to 0.2–3.4 (preprints) indicated a decrease in transmission in Wuhan and other parts of China (*19*,*26*; T. Liu et al., unpub data, https://www.biorxiv.org/content/10.1101/2020.01.25.919787v2; K. Mizumoto et al., unpub. data, https://www.medrxiv.org/content/10.1101/2020.02.12.20022434v1.full.pdf; C. You et al., unpub. data, https://www.medrxiv.org/content/10.1101/2020.02.08.20021253v2.full.pdf; L.Zhang et al., unpub. data, https://www.medrxiv.org/content/10.1101/2020.02.16.20023804v1; C.C. Ku et al., unpub. data, https://papers.ssrn.com/sol3/papers.cfm?abstract_id=3543589; C. Wang et al., unpub. data, https://www.medrxiv.org/content/10.1101/2020.03.03.20030593v1.full.pdf; K.C. Chong et al., unpub. data, https://www.medrxiv.org/content/10.1101/2020.03.02.20028704v1.full.pdf; D. Chen and T. Zhou, unpub data, https://arxiv.org/pdf/2003.00305v1.pdf). In Singapore and South Korea, declines in *R* estimates also suggest decreases in transmission: from 1.1 to 0.7 as of February 14 in Singapore, and 1.5 (95% CI 1.4–1.6) in South Korea up to February 27 (*28*; A. Tariq et al., unpub. data, https://www.medrxiv.org/content/10.1101/2020.02.21.20026435v6.full.pdf). The R estimate for the Diamond Princess cruise ship suggests high transmission before and immediately after movement restrictions on the ship (median R 12.1 [95% CrI 8.2–17.2] on February 7, 2 days postquarantine), with rapid decrease thereafter (median R 0.35 [95% CrI 0.02–2.19] as of February 18) (*29*). Together, these estimates suggest that R_0_ is high, yet intensive interventions can reduce transmissibility (R) substantially.

### Incubation Period

**Figure 2:**
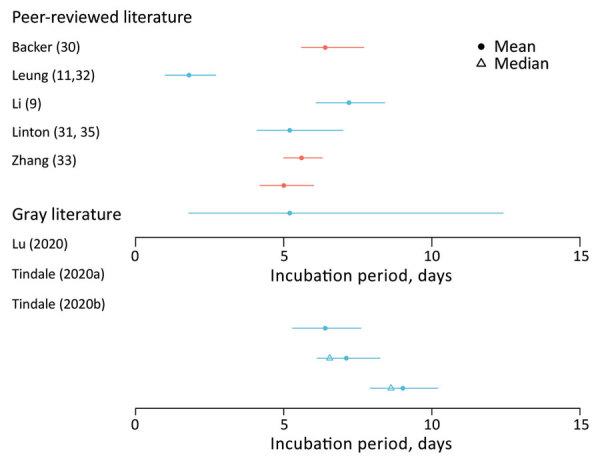
Estimated incubation period for coronavirus disease based on search in peer-reviewed and gray literature. Error bars indicate confidence (blue) or credible (red) intervals. Gray literature sources: Lu et al., unpub. data, https://www.medrxiv.org/content/10.1101/2020.02.19.20025031v1, Tindale et al., unpub. data, https://www.medrxiv.org/content/10.1101/2020.03.03.20029983v1 (also see Appendix Tables 2, 3).

The incubation period is the time between infection and symptom onset. Seven studies (10 estimates) were included in this review; the overall range was 1.8–9.0 days (Figure 2; Appendix Tables 2, 3) (*9*,*30*–*33*; L. Tindale et al., unpub. data, https://www.medrxiv.org/content/10.1101/2020.03.03.20029983v1; H. Lu et al., unpub. data, https://www.medrxiv.org/content/10.1101/2020.02.19.20025031v1). Among the articles in peer-reviewed literature, the mean incubation period was 1.8–6.9 days (*9*,*30*–*33*).

### Serial Interval

**Figure 3:**
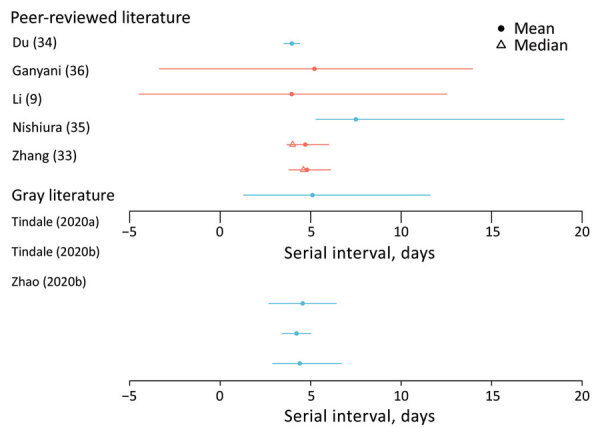
Estimated serial interval for coronavirus disease based on search in peer-reviewed and gray literature. Error bars indicate confidence (blue) or credible (red) intervals. Gray literature sources: Tindale et al., unpub. data, https://www.medrxiv.org/content/10.1101/2020.03.03.20029983v1, Zhao et al., unpub. data, https://www.medrxiv.org/content/10.1101/2020.02.21.20026559v1 (also see Appendix Tables 2, 3).

The serial interval is the average time between symptom onset of a primary and transmission associated secondary case. Seven studies (10 estimates) estimated the mean serial interval in the range of 4.0–7.5 days (Figure 3; Appendix Tables 2, 3) (*9*,*33*–*36*; L. Tindale et al., unpub. data, https://www.medrxiv.org/content/10.1101/2020.03.03.20029983v1; S. Zhao et al., unpub. data, https://www.medrxiv.org/content/10.1101/2020.02.21.20026559v1). Ganyani et al. estimated a mean generation interval of 5.21 in Singapore and 3.95 in Tianjin, China (*36*).

### Doubling Time

**Figure 4:**
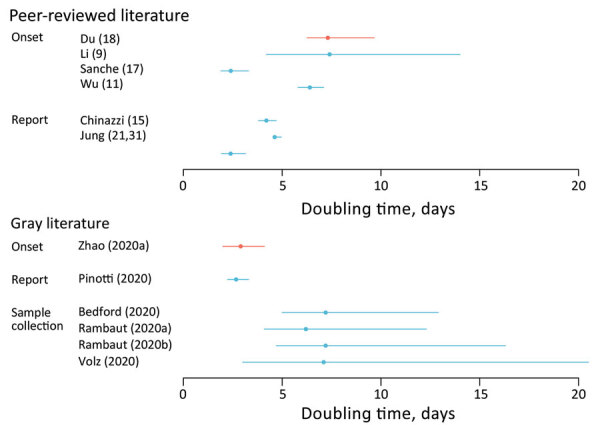
Estimated doubling time for coronavirus disease based on search in peer-reviewed literature and gray literature. Error bars indicate confidence (blue) or credible (red) intervals. Gray literature sources: Onset: Zhao et al., unpub. data, https://www.medrxiv.org/content/10.1101/2020.02.06.20020941v1 ; report: Pinotti et al., unpub. data, https://www.medrxiv.org/content/10.1101/2020.02.24.20027326v1 ; sample collection: Bedford, unpub. data, http://virological.org/t/phylodynamic-estimation-of-incidence-and-prevalence-of-novel-coronavirus-ncov-infections-through-time/3 , Rambaut, unpub. data, http://virological.org/t/phylodynamic-analysis-176-genomes-6-mar-2020/356 , Rambaut, unpub. data, http://virological.org/t/phylodynamic-analysis-176-genomes-6-mar-2020/356 (same) , Volz et al., https://spiral.imperial.ac.uk/bitstream/10044/1/77169/11/2020-02-15-COVID19-Report-5.pdf (also see Appendix Tables 2, 3).

The doubling time is the average time for the daily case count to double. Using both genetic and case data over several locations and time periods, 11 studies estimated a mean doubling time of 2.3–7.4 days (Figure 4, Appendix Tables 2,3) (*9*,*11*,*15*,*17*,*18*,*21*; A. Rambaut, unpub. data, http://virological.org/t/phylodynamic-analysis-176-genomes-6-mar-2020/356; T. Bedford, unpub. data, http://virological.org/t/phylodynamic-estimation-of-incidence-and-prevalence-of-novel-coronavirus-ncov-infections-through-time/391; F. Pinotti et al., unpub. data, https://www.medrxiv.org/content/10.1101/2020.02.24.20027326v1; S. Zhao et al., unpub. data, https://www.medrxiv.org/content/10.1101/2020.02.06.20020941v1; Volz et al., unpub. data, https://spiral.imperial.ac.uk/bitstream/10044/1/77169/11/2020-02-15-COVID19-Report-5.pdf).

### Infectious Period

The infectious period is the period of time in which an infected host, with or without symptoms, can transmit to susceptible persons. One estimate (C. You et al., unpub. data, https://www.medrxiv.org/content/10.1101/2020.02.08.20021253v2), based on data from 67 cases, estimated a mean infectious period of 10.91 days (SD 3.95 days). Little is known about how characteristics of an infected person, such as age, severity, and clinical progression, affect overall infectious period estimates.

### Severity

We did not identify mathematical or statistical models that examine clinical disease progression. We include empiric findings detailed in the WHO China Mission report, which has been used to inform other models (*37*).

**Figure 5:**
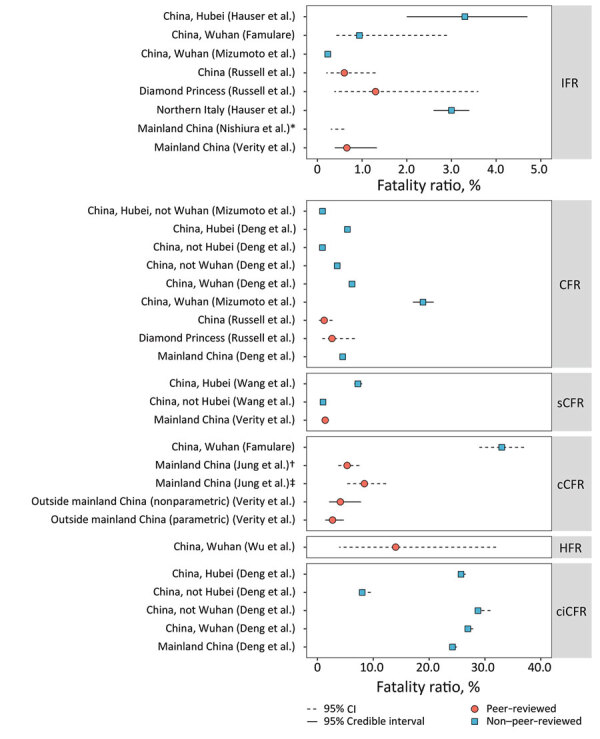
Summary of IFR and CFR estimates for coronavirus disease. Circles or squares indicate mean or median estimates and error bars indicate confidence (dotted line) or credible (full line) intervals. Red indicates peer-reviewed and blue non–peer-reviewed papers (for links to non–peer reviewed papers, see Appendix Table 5). *Range based on »10% ascertainment. †Epidemic growth alone. ‡Epidemic growth along with other parameters. CFR, case fatality ratio; cCFR, laboratory-confirmed CFR; ccCFR, critical care and severe CFR; sCFR, symptomatic CFR; HFR, hospitalization fatality ratio; IFR, infection fatality ratio.

Most of the >75,000 cases of COVID-19 reported through March 6 were from Hubei Province. Among 55,924 confirmed cases in China as of February 20, the median patient age was 51 years (range 2 days–100 years); most (77.8%) patients were 30–69 years of age. The clinical distribution was 80.4% mild/moderate, 13.8% severe, and 6.1% critically ill (Appendix Table 4). Only 2.4% of reported cases were among persons <19 years of age (*37*). Severe disease was reported among those with increased age (>60 years) and underlying conditions such as hypertension, diabetes, cardiovascular disease, chronic respiratory disease, and cancer (*38*). Fatality estimates have come primarily from elderly Wuhan residents (*39*), suggesting substantially higher lethality compared with cases outside Hubei Province (Figure 5).

The age distribution of cases and deaths detected outside China has been wider than that within China (*39*). This difference may result from higher-sensitivity surveillance for travelers compared with cases inside China, particularly in countries on high alert, such as Thailand and Japan, which implemented temperature screening at airports. In general, early severe cases were more likely to be detected than mild cases, resulting in higher severity estimates early on. Cases among travelers might also generally be in younger persons because of age-specific differences in travel.

A broader spectrum of clinical severity has been observed in travel-associated and locally acquired cases reported outside China, likely reflecting more robust surveillance for SARS-CoV-2. Severity ranges from asymptomatic infection to symptoms such as fever and fatigue, as well as mild to severe respiratory symptoms including cough and pneumonia. Cases have been reported in persons with previously good health and no known underlying conditions (*40*). Differences in severity have also been observed within transmission chains (*41*–*44*).

The case-fatality ratio (CFR) is the proportion of cases that result in death. There are several variations of CFR, including symptomatic (sCFR), laboratory-confirmed (cCFR), hospitalization (HFR), critical care (ccCFR), and infection (IFR). Eleven studies, estimating either CFR or a variation of CFR, were included in this review (Figure 5). Most estimates were based on data from China; however, a few are from outside China or from the Diamond Princess cruise ship (*39*,*45*). Estimates of CFR generally did not include specific case definitions and ranged from 0.9% to 18.9%. Moreover, CFR is highly variable across situations (e.g., general population, hospitalized patients, or critically ill patients). Critically ill patients’ estimates range between 8.0% and 28.7% (X. Deng et al., unpub. data, https://www.medrxiv.org/content/10.1101/2020.03.04.20031005v1). Notably, IFR seems to be more consistent across studies, with central estimates around 0.6% in 2 peer-reviewed studies from mainland China (*39*,*45*), yet higher at 3.3% in Hubei Province and 3% in northern Italy (A. Hauser et al., unpub. data, https://www.medrxiv.org/content/medrxiv/early/2020/03/30/2020.03.04.20031104.full.pdf), and lower at 0.2%–1.6% in Asia and Europe (Figure 5; Appendix Table 5).

There was evidence of a strong age gradient in both CFR and IFR; elderly patients were at higher risk (*43*). IFR shows a strong age gradient; IFR was 0.007% in children, 1.9%–4.6% in patients 60–69 years of age, and 7.8%–18% in patients >80 years of age (*39*). Hospitalization rates were also age dependent: <0.04% in children, 11.8% in patients 60–69 years of age, and 18.4% among patients >80 years of age (*39*).

### Viral Evolution and Genomic Epidemiology

Virus genome sequences from a representative sample of cases can be used for calculating the evolutionary rate, date of introduction to the human population, and size of outbreak, as well as estimating the reproduction number and doubling time (*46*–*49*). The evolutionary rate is the rate at which mutations accumulate per base pair in the genome over the course of a year. Estimates have ranged from 0.8 × 10^−3^ to 1.2 × 10^−3^ (Table 2; J. Sciré et al., unpub. data, http://virological.org/t/update-2-evolutionary-epidemiological-analysis-of-128-genomes/423; S. Duchene et al., unpub. data, http://virological.org/t/temporal-signal-and-the-evolutionary-rate-of-2019-n-cov-using-47-genomes-collected-by-feb-01-2020/379; V. Hill and A. Rambaut, unpub. data, https://virological.org/t/phylodynamic-analysis-of-sars-cov-2-update-2020-03-06/420; A. Rambaut, unpub. data, http://virological.org/t/phylodynamic-analysis-176-genomes-6-mar-2020/356; T. Bedford, unpub. data, http://virological.org/t/phylodynamic-estimation-of-incidence-and-prevalence-of-novel-coronavirus-ncov-infections-through-time/391). These evolutionary rates are similar to those of MERS-CoV and SARS-CoV-1. The data suggest that the COVID-19 outbreak was started by a single spillover event occurring in late 2019 (Table 2) and supported by case-reported data in December 2019 (*50*; J. Sciré et al., unpub. data, http://virological.org/t/update-2-evolutionary-epidemiological-analysis-of-128-genomes/423; S. Duchene et al., unpub. data, http://virological.org/t/temporal-signal-and-the-evolutionary-rate-of-2019-n-cov-using-47-genomes-collected-by-feb-01-2020/379; V. Hill and A. Rambaut, unpub. data, http://virological.org/t/phylodynamic-analysis-of-sars-cov-2-update-2020-03-06/420; A. Rambaut, unpub. data, http://virological.org/t/phylodynamic-analysis-176-genomes-6-mar-2020/356; T. Bedford, unpub. data, http://virological.org/t/phylodynamic-estimation-of-incidence-and-prevalence-of-novel-coronavirus-ncov-infections-through-time/391; Volz et al., unpub. data, https://spiral.imperial.ac.uk/bitstream/10044/1/77169/11/2020-02-15-COVID19-Report-5.pdf). 

**Table 2:** Summary of estimates of mean evolutionary rate and most recent common ancestor of COVID-19*

Mean evolutionary rate (95% CI)	MRCA (95% CI)	No. genomes analyzed	Clock model†	Growth model	Source
NA	2019 Nov 29 (Nov 8–Dec 16)	23	Strict	Constant	Rambaut, unpub. data, http://virological.org/t/phylogenetic-analysis-of-23-ncov-2019-genomes-2020-01-23/335
1.23 × 10^−3^ (0.56 × 10^−3^ to 1.98 × 10^−3^)	2019 Nov 21 (Oct 23–Dec 13)	51	Strict	Exponential	Duchene et al., unpub. data, http://virological.org/t/temporal-signal-and-the-evolutionary-rate-of-2019-n-cov-using-47-genomes-collected-by-feb-01-2020/379
1.29 × 10^−3^ (0.535 × 10^−4^ to 2.15 × 10^−3^)	2019 Nov 14 (Sep 28–Dec 13)	51	UNCL	Exponential	Duchene et al., unpub. data, http://virological.org/t/temporal-signal-and-the-evolutionary-rate-of-2019-n-cov-using-47-genomes-collected-by-feb-01-2020/379
0.9 × 10^−3^ (0.5 × 10^−3^ to 1.4 × 10^−3^)	2019 Dec 3 (Oct 30–Dec 17)	51	Strict	Exponential	Bedford, unpub. data, http://virological.org/t/phylodynamic-estimation-of-incidence-and-prevalence-of-novel-coronavirus-ncov-infections-through-time/391
0.92 × 10^−3^ (0.33 × 10^−3^ to 1.46×10^−3^)	2019 Nov 29 (Oct 28–Dec 20)	75	Strict	Exponential	Rambaut, unpub. data, http://virological.org/t/phylodynamic-analysis-176-genomes-6-mar-2020/356. Accessed March 4, 2020
1.04 × 10^−3^ (0.71 × 10^−3^ to 1. 4 ×10^−3^)	2019 Dec 3 (Nov 16–Dec 17)	116	Strict	Exponential	Hill and Rambaut, unpub. data, http://virological.org/t/phylodynamic-analysis-of-sars-cov-2-update-2020-03-06/420
7.41×10^−4^ (4.91 × 10^−4^ to 1.02 × 10^−3^)	2019 Nov 27 (Nov 7–Dec 11)	128	Strict	Birth–death model	Sciré et al., unpub. data, http://virological.org/t/update-2-evolutionary-epidemiological-analysis-of-128-genomes/423

*MRCA, most recent common ancestor; NA, not applicable; UNCL, uncorrelated. †The clock model is a technique that uses the mutation rate to estimate the time of emergence (*48*).

### Effectiveness of Nonpharmaceutical Interventions 

Nonpharmaceutical interventions (NPIs) include interventions at individual and community levels. At the individual level, NPIs examined in modeling studies included voluntary home isolation or quarantine (Appendix Table 6). At the community level, NPIs included school and workplace closures and canceling or postponing large public gatherings (see Appendix Table 7 for definitions). Modeling can be used to estimate the effectiveness of components of these interventions (e.g., case detection), the interventions themselves (e.g., case isolation) or combinations of interventions (e.g., case and contact isolation). In total, 29 articles were identified; of these, 17 met the inclusion criteria for this review (Table 3) (*11*,*15*,*18*,*51*–*56*; F. Pinotti et al., unpub. data, https://www.medrxiv.org/content/10.1101/2020.02.24.20027326v1; R. Niehus et al., unpub. data, K. Gostic et al., unpub. data, https://www.medrxiv.org/content/10.1101/2020.01.28.20019224v2; A. Adiga et al., unpub. data, https://www.medrxiv.org/content/10.1101/2020.02.20.20025882v2; S. Lai et al., unpub. data, https://www.medrxiv.org/content/10.1101/2020.03.03.20029843v3.full.pdf; Y. Zhang et al., unpub. data, https://www.medrxiv.org/content/10.1101/2020.03.04.20031187v1; S. Clifford et al., unpub. data, https://cmmid.github.io/topics/covid19/screening-outbreak-delay.html; S. Bhatia et al., unpub. data, https://www.imperial.ac.uk/media/imperial-college/medicine/sph/ide/gida-fellowships/Imperial-College-COVID19-international-surveillance-21-02-2020.pdf).

**Table 3:** Summary of studies of NPIs for COVID-19

NPI	Summary/results	Source/reference
Case detection	(27%–37%) cases detected†	Bhatia et al., unpub. data, https://www.imperial.ac.uk/media/imperial-college/medicine/sph/ide/gida-fellowships/Imperial-College-COVID19-international-surveillance-21-02-2020.pdf
Case detection	38% (22%–64%) cases detected	Niehus et al., unpub. data, https://www.medrxiv.org/content/10.1101/2020.02.13.20022707v2
Case screening and detection	(36%–65%) cases detected†	Pinotti et al., unpub. data, https://www.medrxiv.org/content/10.1101/2020.02.24.20027326v1
Case isolation and contact tracing	Delay of symptom onset to isolation has a high impact on the results, affecting the controllability of the outbreak. Results vary by scenario.	(*51*)
Travel screening	34% (20%–50%) of travelers identified through both departure and arrival screening using symptoms or risk screening	Gostic et al., unpub. data, https://www.medrxiv.org/content/10.1101/2020.01.28.20019224v2
Travel screening	46.5% (35.9%–57.7%) travelers not detected through thermal screening	(*52*)
Travel screening	Syndromic screening and traveler sensitization in combination could delay outbreaks in yet unaffected countries up to 83 d (75% 36 d, 97.5% 8 d).	Clifford et al., unpub. data, https://cmmid.github.io/topics/covid19/screening-outbreak-delay.html
Travel reduction (transport suspension)	Delay of 2.91 d (95% CI 2.54–3.29) for the arrival of the disease to other cities in China	(*53*)
Travel reduction (travel quarantine)	130 cities in China had >50% chance of having a COVID-19 case imported from Wuhan in the 3 weeks preceding the quarantine.	(*18*)
Travel restrictions	Travel restriction imposed on Wuhan delay the epidemic for 3 d.	(*15*)
Travel reduction (airline suspensions)	Travel restriction imposed on China will delay the disease in other countries, the biggest delay being in Africa (11 d) and South America (9 d).	Adiga et al., unpub. data, https://www.medrxiv.org/content/10.1101/2020.02.20.20025882v2
Travel reduction	Travel restriction will delay the epidemic for 2 d.	(*54*)
Cancellation of mass gathering	37% fewer cases when the interventions started before the first case	(*53*)
Combination of NPI	66%, 86%, and 95% fewer cases depending on timing of the interventions	Lai et al., upub. data, https://www.medrxiv.org/content/10.1101/2020.03.03.20029843v3.full.pdf
Combination of NPI	50% fewer cases if transmissibility reduced by 25% in all cities in China; delay of epidemic peak for 1 month	(*11*)
Combination of NPI	Drastic control measures implemented in China have substantially mitigated spread of COVID-19.	(*36*)
Combination of NPI	Earlier intervention of social distancing could limit the epidemic in mainland China. Number of infections could be reduced up to 98.9%, and number of deaths could be reduced by up to 99.3% as of Feb 23, 2020.	Zhang et al., unpub. data, https://www.medrxiv.org/content/10.1101/2020.03.04.20031187v1
Community behavior modification	At least 42% of persons interviewed have modified daily behavior.	(*55*)

*COVID-19, coronavirus disease; NPI, nonpharmaceutical interventions. †Point estimates

### Case Screening and Detection

Recent articles have addressed the efficacy of screening and detection by surveillance systems in different countries (*51*; F. Pinotti et al., unpub. data, https://www.medrxiv.org/content/10.1101/2020.02.24.20027326v1; R. Niehus et al., unpub. data, https://www.medrxiv.org/content/10.1101/2020.02.13.20022707v2; S. Bhatia et al., unpub. datahttps://www.imperial.ac.uk/media/imperial-college/medicine/sph/ide/gida-fellowships/Imperial-College-COVID19-international-surveillance-21-02-2020.pdf). Two studies used data from Singapore (known for having a reliable health reporting system) as benchmarks to estimate the sensitivity of surveillance systems in other countries (R. Niehus et al., unpub. data, https://www.medrxiv.org/content/10.1101/2020.02.13.20022707v2; S. Bhatia et al., unpub. data,https://www.imperial.ac.uk/media/imperial-college/medicine/sph/ide/gida-fellowships/Imperial-College-COVID19-international-surveillance-21-02-2020.pdf). Both articles agreed that only a fraction of cases (22%–64%) are captured by surveillance systems, varying by country. A more recent study found similar results (36% detected cases), and lower ascertainment when repatriations were considered (F. Pinotti et al., unpub. data, https://www.medrxiv.org/content/10.1101/2020.02.24.20027326v1).

### Case Isolation and Quarantine of Contacts

One study considered different scenarios in which the reproduction number and transmission before symptom onset were varied to study the controllability of the outbreak (*51*). The authors found that as R_0_ increased, the percentage of contacts to be traced increased. The delay between symptom onset and isolation also affected the controllability of the outbreak. For values of R_0_ >2.5, contact tracing and isolation were successful at stopping transmission when <1% of transmission occurred before symptom onset. For these 2 parameters, case isolation alone would be unlikely to control transmission within 3 months.

### Traveler Screening

Two studies considered models in which passengers were screened before leaving an area with local transmission and upon arrival at their destination (*52*; K. Gostic et al., unpub. data, https://www.medrxiv.org/content/10.1101/2020.01.28.20019224v2), demonstrating that a relatively low number of cases would likely be detected (34%–54%). Different factors affect the underdetection of cases, including the country’s ability to detect cases. Some of those factors include asymptomatic infections, infections with mild clinical symptoms, limited care-seeking behavior, case definition, and underrecognition of cases by clinicians. A third study suggests that exit and entry screening combined with traveler sensitization can delay a local outbreak by ≥83 days, with <1 infected traveler per week (S. Clifford et al., unpub. data, https://cmmid.github.io/topics/covid19/screening-outbreak-delay.html).

### Travel Restrictions

On January 23, 2020, travel bans were implemented from Wuhan city. Within China, this resulted in a delay in disease arrival of 3 days, on average (*53*). Cities that implemented the ban before their first case was detected observed fewer cases than cities that implemented the ban after their first case (*53*). Another study found that 130 cities in China had >50% chance of having a COVID-19 case imported from Wuhan in the 3 weeks preceding the implementation of travel restrictions, suggesting that there were cases outside of Wuhan before the travel ban (*18*). Analysis of the effect of the Wuhan travel ban, including the implementation of long-range travel restrictions on January 23, showed no noticeable difference for the epidemic trajectory of Wuhan, while delaying the occurrence of cases for other locations in China by 3 days (*15*). Another study found that travel restrictions would delay the epidemic spread throughout China by 2 days (*54*).

Internationally, several countries implemented travel bans. One modeling study estimated how travel restrictions from China affected the time of arrival of the infected persons (A. Adiga et al., unpub. data, https://www.medrxiv.org/content/10.1101/2020.02.20.20025882v2). It found that countries in Africa and South America would likely observe the biggest delays: 11 days for Africa and 9 days for South America. Another study found that travel reductions of up to 90% had only a modest effect unless paired with public health interventions and behavioral changes to achieve a considerable reduction in disease transmission (*15*).

### Cancellation of Events and Public Gatherings

One study analyzed a range of interventions, such as suspending public transport, closing entertainment venues, and banning public gatherings (*53*). Results varied by city and number of control measures adopted; the study found that cities that implemented a level 1 response (>2 control measures) before the first case was confirmed had 37% fewer cases in the week after the first case identified compared with cities that started control thereafter. Locations that closed entertainment venues and banned public gatherings early in the outbreak reported fewer cases during the first week.

Finally, 4 studies estimated the effects of transmission reduction in China when NPI mitigation strategies were combined (*11*; S. Lai et al., unpub. data, https://www.medrxiv.org/content/10.1101/2020.03.03.20029843v3.full.pdf). The combined interventions notably reduced the number of cases observed and delayed the epidemic peak by >1 month. It was found that earlier intervention of social distancing could greatly limit the epidemic in mainland China. The number of infections could have been reduced up to 98.9%, and the number of deaths reduced by 99.3%, as of February 23, 2020 (Y. Zhang et al., unpub. data, https://www.medrxiv.org/content/10.1101/2020.03.04.20031187v1). A different group found that following the implementation of control measures, growth rates became negative in most locations, and that drastic control measures implemented in China substantially mitigated COVID-19 spread (*56*).

### Community Behavior Modification

One research group performed an online survey after the first case of COVID-19 was reported in Hong Kong. Those results showed that 39%–88% of the persons surveyed had adopted social distancing measures (*55*).

## Discussion

Modeling can provide estimates of disease transmission parameters for planning and response during epidemics. Investigators around the world have been trying to understand the transmission dynamics and severity of disease, as well as the effects that different interventions have had on the course of the epidemic through advanced analytics and modeling. However, transmission parameter estimates are limited by the availability and comprehensiveness of data early in the epidemic. Some parameters can be estimated from genetic sequencing data, but these estimates are heavily influenced by biases in sampling and inaccuracies in sequencing. Although efforts to collect and share clinical, epidemiologic, and sequence data have been remarkably timely, there remain outstanding gaps in knowledge.

Several parameters presented in this review are context specific, such as R_0_ values or CFR measurements. Although the characteristics of SAR-CoV-2 are unlikely to change, responses to transmission will vary. Several factors affect the trajectory of an epidemic in different locations, such as population density, health system infrastructure, transportation robustness, cultural practices, and poverty levels (*57*). Available data from China may not be reflective of secular trends elsewhere. As other countries develop more cases, more robust data will be available for modeling and extrapolation for countries not yet affected.

Challenges in assessing severity of clinical outcomes during a new emerging epidemic have been discussed in depth elsewhere and are not covered here (*7*,*8*). However, 4 challenges remain. First, early in an outbreak, data are heavily biased toward severe cases. Estimates of the CFR in those patients with known outcomes may be biased upward until the extent of clinically milder disease is determined. Second, there is a period between onset of symptoms and final clinical outcome (death vs. survival) (*58*,*59*). During a growing epidemic, the final clinical outcome of most reported cases is typically unknown. This is particularly true with COVID-19, with which severely ill patients may be hospitalized for many days. The crude CFR will underestimate the fatality risk among early epidemic cases (*7*,*8*,*59*). Third, while the epidemic is growing there will be a bias toward having observed cases with recent symptom onset and outcomes. Therefore, estimates should be adjusted for the growth rate of the epidemic (*8*). Fourth, overrepresentation of men, elderly persons with underlying conditions, and persons with respiratory risk factors (such as smoking) may result from observation bias or exposure differences and affect CFR estimates.

Country preparedness and clinical care capacity will affect patient outcomes. Delayed diagnosis and treatment, limited knowledge of the natural history of infection, and rapid escalation of cases can affect clinical outcomes. Thus, fatality in patients cared for very early in a country’s epidemic may be greater than in later patients (*7*). More information on the proportion of persons requiring healthcare, level of care (outpatient, inpatient, and intensive care), and duration of care required are essential for predicting healthcare needs as the epidemic progresses.

Presymptomatic or asymptomatic transmission, if substantial, might have critical implications for control efforts. Empiric evidence of such potential transmission includes a serial interval and generation time that were estimated shorter than the incubation period (L. Tindale et al., unpub.data, https://www.medrxiv.org/content/10.1101/2020.03.03.20029983v1; H. Lu et al., unpub. data, https://www.medrxiv.org/content/10.1101/2020.02.19.20025031v1), similarly high viral loads in asymptomatic and symptomatic cases (*36*,*60*), and documentation of patients infected by presymptomatic or asymptomatic carriers in cluster investigations (*61*–*64*). If asymptomatic infectious carriers are not characterized appropriately in models, epidemic infection rates would be underestimated but the severity and the effectiveness of interventions would be overestimated, potentially leading to implementation of ineffective interventions. Serologic studies will be critical for understanding the role of asymptomatic transmission.

Early evidence suggests that travel restrictions result in only modest decreases in the importation of cases. However, combined with other social distancing measures and behavior changes, travel restrictions may be a useful addition. Modeling can be extremely valuable in providing counterfactuals aimed at disentangling the effects of different NPIs. Documentation of timing, type of NPI, and compliance rate will be needed to estimate the effectiveness of the different interventions.

This study is subject to additional limitations. To use the latest information, we included a number of preprint reports that have not been formally peer reviewed. In addition, there is a heavy reliance on data from China, because of the period considered. Given the recent geographic spread of COVID-19, there may be a range of future estimates that will differ from those reported here. Finally, we have not performed a formal assessment of possible biases of the estimates examined in this article, and therefore cannot exclude that some estimates reported are affected by unmeasured sources of biases.

As the COVID-19 epidemic progresses, ongoing refinement and validation of key epidemiologic parameters will help inform the global public health response. Defining optimal surveillance methods, laboratory testing, contact tracing parameters, quarantine measures, hospital acute care capacities, and many other operational factors depends on estimates of the epidemiologic parameters summarized in this article. One of the largest knowledge gaps is that of asymptomatic or presymptomatic infectious potential and the occurrence of subclinical infections. In the absence of efficacious vaccines and therapeutics, developing an evidence base for NPIs will remain a critical tool for effective local, national, and global outbreak control. Better data will enable mathematical and statistical modeling to more precisely predict how different NPIs can be combined to produce efficient epidemic control.

Our summary provides estimates through the first 10 weeks of the COVID-19 epidemic that are needed for operational planning, scenario-building for contingency planning, and forecasting to inform today’s preparedness and response efforts. Data from outbreaks in newly affected countries and new data stemming from seroprevalence and transmission studies will provide insights currently unavailable. Documenting and evaluating NPIs will help public health and government decision makers to implement the most effective epidemic control measures.

Appendix Additional information about insights from statistical and mathematical modeling of key epidemiologic parameters of COVID-19.
