# Supplementary material for: Early Insights from Statistical and Mathematical Modeling of Key Epidemiologic Parameters of COVID-19
Source: Emerg Infect Dis. 2020 Nov;26(11):e201074. doi: 10.3201/eid2611.201074 (PMC7588530; doi:10.3201/eid2611.201074)
Supplement: Appendix — Additional information about insights from statistical and mathematical modeling of key epidemiologic parameters of COVID-19. [file 20-1074-Techapp-s1.pdf]

Article DOI: https://doi.org/10.3201/eid2611.201074

# Early Insights from Statistical and Mathematical Modeling of Key Epidemiologic Parameters of COVID-19

## Appendix

**Appendix Table 1.** Search terms in study of modeling of key epidemiologic parameters of COVID-19

| Topic | Search terms |
|---|---|
| | "nCoV," "2019-nCoV," "nCoV-2019," "COVID," "COVID-19," "novel coronavirus," "SARS-CoV-2"<br>AND<br>"evolution*" or "phylogenetics" |
| Incubation period | In PubMed, medRxiv, bioRxiv and arXiv:<br>#1: "incubation period"<br>#2: "coronavirus" OR "nCoV" OR "COVID" OR "COVID-19" OR "2019-nCoV" OR "nCoV-2019" OR "SARS-CoV-2"<br>#3: #1 AND #2<br>In SSRN:<br>#1: "incubation period"<br>#2: "coronavirus" OR "nCoV" OR "COVID" OR "COVID-19" OR "2019-nCoV" OR "nCoV-2019" OR "SARS-CoV-2"<br>#3: #1 AND search within each of the term in #2<br>In research square, Virological, and Wellcome Open Research:<br>#1: "coronavirus" OR "nCoV" OR "COVID" OR "COVID-19" OR "2019-nCoV" OR "nCoV-2019" OR "SARS-CoV-2"<br>Then read through all the papers |
| Serial interval | In PubMed:<br>#1: "serial interval" OR "generation interval" OR "generation time" OR "serial distribution" OR "secondary infections" OR "secondary cases"<br>#2: "coronavirus" OR "nCoV" OR "COVID" OR "COVID-19" OR "2019-nCoV" OR "nCoV-2019" OR "SARS-CoV-2"<br>#3: #1 AND #2<br>In medRxiv, bioRxiv, and arXiv:<br>#1: "serial interval"<br>#2: "coronavirus" OR "nCoV" OR "COVID" OR "COVID-19" OR "2019-nCoV" OR "nCoV-2019" OR "SARS-CoV-2"<br>#3: #1 AND #2<br>In SSRN:<br>#1: "serial interval"<br>#2: "coronavirus" OR "nCoV" OR "COVID" OR "COVID-19" OR "2019-nCoV" OR "nCoV-2019" OR "SARS-CoV-2"<br>#3: #1 AND search within each of the term in #2<br>In research square, Virological, and Wellcome Open Research:<br>#1: "coronavirus" OR "nCoV" OR "COVID" OR "COVID-19" OR "2019-nCoV" OR "nCoV-2019" OR "SARS-CoV-2"<br>Then read through all the papers |
| Generation interval | In PubMed and arXiv:<br>#1: "generation interval"<br>#2: "coronavirus" OR "nCoV" OR "COVID" OR "COVID-19" OR "2019-nCoV" OR "nCoV-2019" OR "SARS-CoV-2"<br>#3: #1 AND #2<br>In medRxiv and bioRxiv: |

| Topic | Search terms |
|---|---|
| | "generation interval" for full text or abstract or title (match whole all)<br>In SSRN:<br>#1: "generation interval"<br>#2: "coronavirus" OR "nCoV" OR "COVID" OR "COVID-19" OR "2019-nCoV" OR "nCoV-2019" OR "SARS-CoV-2"<br>#3: #1 AND search within each of the term in #2<br>In research square, Virological, and Wellcome Open Research:<br>#1: "coronavirus" OR "nCoV" OR "COVID" OR "COVID-19" OR "2019-nCoV" OR "nCoV-2019" OR "SARS-CoV-2"<br>Then read through all the papers |
| Doubling time | In PubMed, medRxiv, bioRxiv and arXiv:<br>#1: "doubling time" OR "growth rate"<br>#2: "coronavirus" OR "nCoV" OR "COVID" OR "COVID-19" OR "2019-nCoV" OR "nCoV-2019" OR "SARS-CoV-2"<br>#3: #1 AND #2<br>In SSRN:<br>#1: "doubling time" OR "growth rate"<br>#2: "coronavirus" OR "nCoV" OR "COVID" OR "COVID-19" OR "2019-nCoV" OR "nCoV-2019" OR "SARS-CoV-2"<br>#3: #1 AND search within each of the term in #2<br>In research square, Virological, and Wellcome Open Research:<br>#1: "coronavirus" OR "nCoV" OR "COVID" OR "COVID-19" OR "2019-nCoV" OR "nCoV-2019" OR "SARS-CoV-2"<br>Then read through all the papers |
| CFR | In PubMed:<br>("coronavirus"[MeSH Terms] OR "coronavirus"[All Fields] OR "2019-ncov"[All Fields] OR "COVID-19"[All Fields] OR "COVID"[All Fields] OR "SARS-COV-2"[All Fields] OR "ncov"[All Fields] OR "ncov-2019"[All Fields]) AND "2019/12/31 00.00"[MHDA]: "2020/03/06 23.59"[MHDA] AND ("fatality"[All Fields] OR "Case Fatality"[All Fields] OR "Infection Fatality"[All Fields])<br>In biorXiv and medrXiv:<br>for title "coronavirus fatality" (match all words) and abstract or title "coronavirus fatality" (match all words) and posted between "01 Jan 2020 and 07 Mar 2020"<br>In arXiv:<br>Query: order: -announced_date_first; size: 50; date_range: from 2020–01–01 to 2020–03–07; include_cross_list: True; terms: AND abstract = coronavirus; AND abstract = fatality<br>In ResearchSquare:<br>SARS-CoV-2 Preprints<br>In Wellcome Research:<br>Query: "coronavirus"<br>In WHO Global Research Database:<br>Query: "sever*"<br>Cited by WHO:<br>Imperial College London, IDO |
| Nonpharmaceutical interventions | In all databases:<br>"coronavirus" OR "nCoV" OR "COVID" OR "COVID-19" OR "2019-nCoV" OR "nCoV- 2019" OR "SARS-CoV-2" |

**Appendix Table 2.** Summary of published studies on COVID-19 included in review of incubation period, serial interval, doubling time, and generation interval*

| Study | Study setting | Incubation period (days) | Serial interval (days) | Doubling time (days) | Generation interval (days) |
|---|---|---|---|---|---|
| Backer 2020 (*1*) | 88 confirmed cases detected outside Wuhan, Jan 20–28, 2020 | Mean 6.4, median NA, SD 2.3, 95% CrI 5.6–7.7 based on Weibull distribution | NA | NA | NA |
| Chinazzi 2020 (*2*) | Modeling study | NA | NA | 4.2 (90% CI 3.8–4.7) based on reporting dates | NA |
| Du 2020a (*3*) | Modeling study | NA | NA | 7.31 (95% CrI 6.26–9.66) based on onset dates | NA |
| Du 2020b (*4*) | 468 confirmed infector–infectee pairs in 18 Chinese provinces, from Jan 21– Feb 8, 2020 | NA | Mean 3.96,median NA,SD 4.75,95% CI 3.53–4.39, based on normal distribution | NA | NA |
| Ganyani 2020 (*5*) | Modeling study | NA | Mean 5.21, median NA, SD 4.32, 95% CrI −3.35 to 13.94 in Singapore | NA | Mean 5.20, median NA, SD 1.72, 95% CrI 3.78–6.78 in Singapore |
| Ganyani 2020 (*5*) | Modeling study | NA | Mean 3.95, median NA, SD 4.24, 95% CrI −4.47 to 12.51 in Tianjin, China | NA | Mean 3.95, median NA, SD 1.51, 95% CrI 3.01–4.91 in Tianjin, China |
| Jung 2020 (*6*) | Modeling study | NA | NA | 4.62 (95% CI 4.62–4.95) from a single index case with illness onset on Dec 8, 2019, calculated by growth rate of 0.15 (95% CI 0.14–0.15) based on reporting dates | NA |
| Jung 2020 (*6*) | Modeling study | NA | NA | 2.39 (95% CI 1.93–3.15) from 20 exported cases reported by Jan 24, 2020, calculated by growth rate of 0.29 (95% CI 0.22–0.36) based on reporting dates | NA |
| Leung 2020 (*7*) | 175 confirmed patients in China, Jan 20–Feb 12, 2020 | Mean 1.8, median NA, SD NA, 95% CI 1.0–2.7 based on travelers to Hubei fitting to Weibull distribution | NA | NA | NA |
| Leung 2020 (*7*) | 175 confirmed patients in China, Jan 20–Feb 12, 2020 | Mean 7.2, median NA, SD NA, 95% CI 6.1–8.4 based on nontravelers fitting to Weibull distribution | NA | NA | NA |
| Li 2020 (*8*) | 425 confirmed cases in Wuhan as of Jan 22, 2020 | Mean 5.2, median NA, SD NA, 95% CI 4.1–7.0 based on log-normal distribution | Mean 7.5, median NA, SD 3.4, 95% CI 5.3–19.0 based on gamma distribution | 7.4 (95% CI 4.2–14.0) based on onset dates | NA |
| Linton 2020 (*9*) | 158 confirmed cases in and outside Wuhan as of Jan 31, 2020 (52 cases when excluding Wuhan residents) | Mean 5.6, median NA, SD NA, 95% CrI 5.0–6.3 based on 158 cases | NA | NA | NA |
| Linton 2020 (*9*) | 158 confirmed cases in and outside Wuhan as of Jan 31, 2020 (52 cases | Mean 5.0, median NA, SD NA, 95% CrI 4.2–6.0 based on 52 cases | NA | NA | NA |

| Study | Study setting | Incubation period (days) | Serial interval (days) | Doubling time (days) | Generation interval (days) |
|---|---|---|---|---|---|
| | when excluding Wuhan residents) | | | | |
| Nishiura 2020 (*10*) | 28 infector–infectee pairs from published research articles as of Feb 12, 2020 | NA | Mean 4.7, median 4.0, SD 2.9, 95% CrI 3.7–6.0 based on all 28 pairs fitting to log-normal distribution | NA | NA |
| Nishiura 2020 (*10*) | 18 pairs with highest certainty from published research articles as of Feb 12, 2020 | NA | Mean 4.8, median 4.6, SD 2.3, 95% CrI 3.8–6.1 based on 18 certain pairs fitting to Weibull distribution | NA | NA |
| Sanche 2020 (*11*) | Modeling study | NA | NA | 2.4 (95% CI 1.9–3.3) based on onset dates | NA |
| Wu 2020 (*12*) | Modeling study | NA | NA | 6.4 (95% CrI 5.8–7.1) based on onset dates | NA |
| Zhang 2020 (*13*) | 8,579 confirmed cases reported outside Hubei in China as of Feb 17, 2020 (only 49 cases with no travel history to/from Wuhan/Hubei) | Mean 5.2, median NA, SD NA, 95% CI 1.8–12.4based on log-normal distribution | Mean 5.1, median NA, SD NA, 95% CI 1.3–11.6 based on gamma distribution | NA | NA |

*CI, confidence interval; CrI, credible interval; NA, not applicable; SD, standard deviation

**Appendix Table 3.** Summary of the COVID-19 studies included in the review of incubation period, serial interval, and doubling time based on search in gray literature*

| Study | Study setting | Incubation period (days) | Serial interval (days) | Doubling time (days) |
|---|---|---|---|---|
| Bedford, unpub. data, http://virological.org/t/phylodynamic-estimation-of-incidence-and-prevalence-of-novel-coronavirus-ncov-infections-through-time/39 | 53 publicly available nCoV genomes collected between Dec 24, 2019–Feb 4, 2020 | NA | NA | 7.2 (95% CI 5.0–12.9) based on sample collection dates |
| Lu et al., unpub. data, https://www.medrxiv.org/content/10.1101/2020.02.19.20025031v1 | 265 confirmed cases in Shanghai before Feb 7, 2020 (only 27 had credible contact information) | Mean 6.4, median NA, SD NA, 95% CI 5.3–7.6 based on Weibull distribution | NA | NA |
| Pinotti et al., unpub. data, https://www.medrxiv.org/content/10.1101/2020.02.24.20027326v1 | Modeling study | NA | NA | 2.67 (95% CI 2.24–3.30) importations from Hubei, calculated by growth rate of 0.26 (95% CI 0.21–0.31) based on reporting dates |
| Rambaut, unpub. data, http://virological.org/t/phylodynamic-analysis-176-genomes-6-mar-2020/356 | 75 genomes on Feb 12, 2020; 86 genomes on Feb 24, 2020 | NA | NA | 6.2 (95% CI 4.1–12.3), 75 genomes based on sample collection dates |
| Rambaut, unpub. data, http://virological.org/t/phylodynamic-analysis-176-genomes-6-mar-2020/356 | 75 genomes on Feb 12, 2020; 86 genomes on Feb 24, 2020 | NA | NA | 7.2 (95% CI 4.7–16.3), 86 genomes based on sample collection dates |
| Tindale et al., unpub. data, https://www.medrxiv.org/content/10.1101/2020.03.03.20029983v1 | 93 confirmed cases in Singapore during Jan 19–Feb 26, 2020 | Mean 7.11, median: 6.55, SD NA, 95% CI 6.13–8.25 based on Weibull distribution | Mean 4.56, median NA, SD 0.95, 95% CI 2.69–6.42 based on expectation-maximization approach | NA |
| Tindale et al., unpub. data, https://www.medrxiv.org/content/10.1101/2020.03.03.20029983v1 | 125 confirmed cases in Tianjin during Jan 21–Feb 22, 2020 | Mean 9.02, median 8.62, SD NA, 95% CI 7.92–10.2 based on Weibull distribution | Mean 4.22, median NA, SD 0.4, 95% CI 3.43–5.01 based on expectation-maximization approach | NA |
| Volz et al., unpub. data, https://spiral.imperial.ac.uk/bitstream/10044/1/77169/11/2020-02-15-COVID19-Report-5.pdf | Phylogenetic analysis of 53 SARS-CoV-2 whole genome sequences | NA | NA | 7.1 (95% CI 3.0–20.5) based on sample collection dates |
| Zhao et al., unpub. data, https://www.medrxiv.org/content/10.1101/2020.02.21.20026559v1 | 21 infector–infectee pairs in Hong Kong during Jan 16–Feb 15, 2020 | NA | Mean 4.4, median NA, SD 3.0, 95% CI 2.9–6.7, based on gamma distribution | NA |
| Zhao et al., unpub. data, https://www.medrxiv.org/content/10.1101/2020.02.06.20020941v1 | Modeling study | NA | NA | 2.9 (95% CrI 2.0–4.1) based on onset dates |

*CI, confidence interval; CrI, credible interval; NA, not applicable; SD, standard deviation

**Appendix Table 4.** Frequency of different case severities

| Classification | Definition | Proportion* |
|---|---|---|
| Mild/moderate | Non-pneumonia and pneumonia cases | 80.9% |
| Severe | Dyspnea, respiratory frequency >30/minute, blood oxygen saturation ≤93%, $PaO_2/FiO_2$ ratio <300, and/or lung infiltrates >50% within 24–48 h | 13.8% |
| Critical | Respiratory failure, septic shock, and/or multiple organ dysfunction/failure | 6.1% |

* Proportion of 55,924 cases until Feb 20, 2020.

**Appendix Table 5.** Summary of CFR and IFR estimate sources

| Author | Location | Metric * | Estimate | Uncertainty type | Peer-reviewed | Source |
|---|---|---|---|---|---|---|
| **Russell et al.** | China | IFR | 0.6 (0.2–1.3) | 95% CI | Yes | https://www.ncbi.nlm.nih.gov/pubmed/32234121 |
| **Hauser et al.** | China, Hubei | IFR | 3.3 (2–4.7) | 95% CrI | No | https://www.medrxiv.org/content/10.1101/2020.03.04.20031104v2.full.pdf |
| **Famulare** | China, Wuhan | IFR | 0.9 (0.4–2.9) | 95% CI | No | https://institutefordiseasemodeling.github.io/nCoV-public/analyses/first_adjusted_mortality_estimates_and_risk_assessment/2019-nCoV-preliminary_age_and_time_adjusted_mortality_rates_and_pandemic_risk_assessment.html |
| **Mizumoto et al.** | China, Wuhan | IFR | 0.2 (0.2–0.3) | 95% CrI | No | https://www.medrxiv.org/content/10.1101/2020.02.12.20022434v1 |
| **Russell et al.** | Diamond Princess | IFR | 1.3 (0.4–3.6) | 95% CI | Yes | https://www.ncbi.nlm.nih.gov/pubmed/32234121 |
| **Hauser et al.** | Northern Italy | IFR | 3 (2.6–3.4) | 95% CrI | No | https://www.medrxiv.org/content/10.1101/2020.03.04.20031104v1.full.pdf |
| **Nishiura et al.†** | Mainland China | IFR | NA (0.3–0.6) | 95% CI | Yes | https://www.mdpi.com/2077-0383/9/2/419 |
| **Verity et al.** | Mainland China | IFR | 0.7 (0.4–1.3) | 95% CrI | Yes | https://www.thelancet.com/journals/laninf/article/PIIS1473-3099(20)30243-7/fulltext |
| **Russell et al.** | China | CFR | 1.2 (0.3–2.7) | 95% CI | Yes | https://www.ncbi.nlm.nih.gov/pubmed/32234121 |
| **Deng et al.** | China, Hubei | CFR | 5.4 (5.3–5.6) | 95% CI | No | https://www.medrxiv.org/content/10.1101/2020.03.04.20031005v1 |
| **Mizumoto et al.** | China, Hubei not Wuhan | CFR | 0.9 (0.6–1.3) | 95% CrI | No | https://www.medrxiv.org/content/10.1101/2020.02.19.20025163v1 |
| **Deng et al.** | China, not Hubei | CFR | 0.9 (0.8–1.1) | 95% CI | No | https://www.medrxiv.org/content/10.1101/2020.03.04.20031005v1 |
| **Deng et al.** | China, not Wuhan | CFR | 3.5 (3.4–3.8) | 95% CI | No | https://www.medrxiv.org/content/10.1101/2020.03.04.20031005v1 |
| **Deng et al.** | China, Wuhan | CFR | 6.2 (6.1–6.4) | 95% CI | No | https://www.medrxiv.org/content/10.1101/2020.03.04.20031005v1 |
| **Mizumoto et al.** | China, Wuhan | CFR | 18.9 (17.1–20.8) | 95% CrI | No | https://www.medrxiv.org/content/10.1101/2020.02.19.20025163v1 |
| **Russell et al.** | Diamond Princess | CFR | 2.6 (0.9–6.7) | 95% CI | Yes | https://www.ncbi.nlm.nih.gov/pubmed/32234121 |
| **Deng et al.** | Mainland China | CFR | 4.5 (4.5–4.7) | 95% CI | No | https://www.medrxiv.org/content/10.1101/2020.03.04.20031005v1 |
| **Wang et al.** | China, Hubei | sCFR | 7.2 (6.6–8) | 95% CI | No | https://www.medrxiv.org/content/10.1101/2020.02.17.20023630v4 |
| **Wang et al.** | China, not Hubei | sCFR | 1 (0.9–1.2) | 95% CI | No | https://www.medrxiv.org/content/10.1101/2020.02.17.20023630v4 |
| **Verity et al.** | Mainland China | sCFR | 1.4 (1.2–1.5) | 95% CrI | Yes | https://www.thelancet.com/journals/laninf/article/PIIS1473-3099(20)30243-7/fulltext |
| **Famulare** | China, Wuhan | cCFR | 33 (29–37) | 95% CI | No | https://institutefordiseasemodeling.github.io/nCoV-public/analyses/first_adjusted_mortality_estimates_and_risk_assessment/2019-nCoV-preliminary_age_and_time_adjusted_mortality_rates_and_pandemic_risk_assessment.html |
| **Jung et al. ‡** | Mainland China | cCFR | 5.3 (3.5–7.5) | 95% CI | Yes | https://www.ncbi.nlm.nih.gov/pubmed/32075152 |
| **Jung et al. §** | Mainland China | cCFR | 8.4 (5.3–12.3) | 95% CI | Yes | https://www.ncbi.nlm.nih.gov/pubmed/32075152 |

| Author | Location | Metric* | Estimate | Uncertainty type | Peer-reviewed | Source |
|---|---|---|---|---|---|---|
| **Verity et al.** | Outside mainland China (non-parametric) | cCFR | 4.1 (2.1–7.8) | 95% CrI | Yes | https://www.thelancet.com/journals/laninf/article/PIIS1473-3099(20)30243-7/fulltext |
| **Verity et al.** | Outside mainland China (parametric) | cCFR | 2.7 (1.4–4.7) | 95% CrI | Yes | https://www.thelancet.com/journals/laninf/article/PIIS1473-3099(20)30243-7/fulltext |
| **Wu et al.** | China, Wuhan | HFR | 14 (3.9–32) | 95% CI | Yes | https://www.eurosurveillance.org/content/10.2807/1560-7917.ES.2020.25.3.2000044 |
| **Deng et al.** | China, Hubei | ccCFR | 25.7 (25.4–26.5) | 95% CI | No | https://www.medrxiv.org/content/10.1101/2020.03.04.20031005v1 |
| **Deng et al.** | China, not Hubei | ccCFR | 8 (7.5–9.6) | 95% CI | No | https://www.medrxiv.org/content/10.1101/2020.03.04.20031005v1 |
| **Deng et al.** | China, not Wuhan | ccCFR | 28.8 (28–31) | 95% CI | No | https://www.medrxiv.org/content/10.1101/2020.03.04.20031005v1 |
| **Deng et al.** | China Wuhan | ccCFR | 26.9 (26.6–27.9) | 95% CI | No | https://www.medrxiv.org/content/10.1101/2020.03.04.20031005v1 |
| **Deng et al.** | mainland China | ccCFR | 24.2 (23.9–25) | 95% CI | No | https://www.medrxiv.org/content/10.1101/2020.03.04.20031005v1 |

***CFR, case fatality ratio; sCFR, symptomatic CFR; cCFR, laboratory-confirmed CFR; ccCFR, critical care and severe CFR; HFR, hospitalization fatality ratio; IFR, infection fatality ratio.**
**†Range based on ≈10% ascertainment.**
**‡Fitted to epidemic growth alone.**
**§Fitted to epidemic growth along with other parameters.**

**Appendix Table 6.** Types of nonpharmaceutical interventions that could be implemented at the individual and community level

| Type | Nonpharmaceutical intervention |
|---|---|
| Individual interventions | Voluntary home isolation: separation of persons ill with contagious diseases from noninfected persons |
| | Voluntary home quarantine: restriction of persons who are presumed to have been exposed to a contagious disease but are not ill, either because they did not become infected or because they are still in the incubation period |
| Community level | School closure (closure of day care facilities, schools, and higher education) |
| | Workplace closure (closure of nonessential services) |
| | Cancel or postpone large public gatherings |

**Appendix Table 7.** Definition of nonpharmaceutical intervention search keywords*

| Topic | Definition |
|---|---|
| Case detection, case detected | Detection of cases imported from an affected area |
| Case isolation and contact tracing | Isolation of an identified positive case. |
| Travel screening’ | Screening of passengers at port of exit/entry |
| Travel reduction, reduced travel, airline suspension | Mobility reduction of persons, intra- or inter-city/country. |
| School closure | Closing schools to prevent further transmission |
| Cancellation of events and mass gatherings, lockdown | Cancellation of events and mass gatherings to prevent further transmission. |
| Community response | Population’s psychological and behavioral responses during an outbreak |

*This table gives a short definition of the search keywords related to nonpharmaceutical intervention we are interested in. Because there are no specific terms to describe these interventions, or there are different terms to describe the same kind of intervention, we considered them to be equivalent.

## References


1. Backer JA, Klinkenberg D, Wallinga J. Incubation period of 2019 novel coronavirus (2019-nCoV) infections among travellers from Wuhan, China, 20–28 January 2020. Euro Surveill. 2020;25:2000062. PubMed https://doi.org/10.2807/1560-7917.ES.2020.25.5.2000062

2. Chinazzi M, Davis JT, Ajelli M, Gioannini C, Litvinova M, Merler S, et al. The effect of travel restrictions on the spread of the 2019 novel coronavirus (COVID-19) outbreak. Science. 2020;368:395–400. https://doi.org/10.1126/science.aba9757

3. Du Z, Wang L, Cauchemez S, Xu X, Wang X, Cowling BJ, et al. Risk for transportation of 2019 novel coronavirus disease from Wuhan to other cities in China. Emerg Infect Dis. 2020;26:1049–52. https://doi.org/10.3201/eid2605.200146

<jrn>4. Du Z, Xu X, Wu Y, Wang L, Cowling BJ, Meyers L. Serial interval of COVID-19 among publicly reported confirmed cases. Emerg Infect Dis. 2020;26:1341–3. https://dx.doi.org/10.3201/eid2606.200357</jrn>

5. Ganyani T, Kremer C, Chen D, Torneri A, Faes C, Wallinga J, et al. Estimating the generation interval for coronavirus disease (COVID-19) based on symptom onset data, March 2020. Eurosurveillance. 2020;25:2000257. https://doi.org/10.2807/1560-7917.ES.2020.25.17.2000257

6. Jung SM, Akhmetzhanov AR, Hayashi K, Linton NM, Yang Y, Yuan B, et al. Real-time estimation of the risk of death from novel coronavirus (COVID-19) infection: inference using exported cases. J Clin Med. 2020;9:523. PubMed https://doi.org/10.3390/jcm9020523

7. Leung C. The difference in the incubation period of 2019 novel coronavirus (SARS-CoV-2) infection between travelers to Hubei and nontravelers: the need for a longer quarantine period. Infect Control Hosp Epidemiol. 2020;41:594–6. PubMed https://doi.org/10.1017/ice.2020.81

8. Li Q, Guan X, Wu P, Wang X, Zhou L, Tong Y, et al. Early transmission dynamics in Wuhan, China, of novel coronavirus–infected pneumonia. N Eng J Med. 2020;382:1199–1207. https://doi.org/10.1056/NEJMoa2001316

9. Linton NM, Kobayashi T, Yang Y, Hayashi K, Akhmetzhanov AR, Jung SM, et al. Incubation period and other epidemiological characteristics of 2019 novel coronavirus infections with right truncation: a statistical analysis of publicly available case data. J Clin Med. 2020;9:538. PubMed https://doi.org/10.3390/jcm9020538

10. Nishiura H, Linton NM, Akhmetzhanov AR. Serial interval of novel coronavirus (COVID-19) infections. Int J Infect Dis. 2020;93:284–6. PubMed https://doi.org/10.1016/j.ijid.2020.02.060

11. Sanche S, Lin YT, Xu C, Romero-Severson E, Hengartner N, Ke R. High contagiousness and rapid spread of severe acute respiratory syndrome coronavirus 2. Emerg Infect Dis. 2020;26:1470–7. https://doi.org/10.3201/eid2607.200282

12. Wu JT, Leung K, Leung GM. Nowcasting and forecasting the potential domestic and international spread of the 2019-nCoV outbreak originating in Wuhan, China: a modelling study. Lancet. 2020;395:689–97. PubMed http://dx.doi.org/10.1016/S0140-6736(20)30260-9

13. Zhang J, Litvinova M, Wang W, Wang Y, Deng X, Chen X, et al. Evolving epidemiology and transmission dynamics of coronavirus disease 2019 outside Hubei province, China: a descriptive and modelling study. Lancet Infect Dis. 2020;20:793–802. https://doi.org/10.1016/S1473-3099(20)30230-9
